# 2-Hydr­oxy-*N*′-(2-hydr­oxy-4-methoxy­benzyl­idene)-3-methyl­benzohydrazide monohydrate

**DOI:** 10.1107/S1600536810016855

**Published:** 2010-05-15

**Authors:** You-Yue Han, Yong-Hong Li, Qiu-Rong Zhao

**Affiliations:** a Department of Chemistry and Life Science, Chuzhou University, Chuzhou, Anhui 239000, People’s Republic of China

## Abstract

In the title compound, C_16_H_16_N_2_O_4_·H_2_O, the dihedral angle between the two benzene rings is 12.4 (2)° and the mol­ecule adopts an *E* configuration with respect to the C=N bond. There are intra­molecular O—H⋯N and O—H⋯O hydrogen bonds in the hydrazone mol­ecule, which both generate *S*(6) rings. In the crystal structure, mol­ecules are linked by N—H⋯O and O—H⋯O hydrogen bonds, forming layers parallel to the *ab* plane. The crystal studied was a non-merohedral twin with a domain ratio of 0.887 (3):0.113 (3).

## Related literature

For our previous studies on hydrazones and for background information, see: Han & Zhao (2010*a* ▶,*b* ▶). For reference bond-length data, see: Allen *et al.* (1987 ▶).
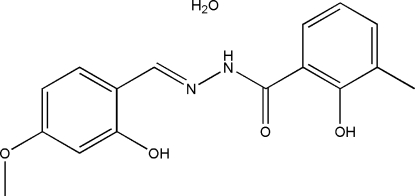


## Experimental

### 

#### Crystal data

C_16_H_16_N_2_O_4_·H_2_O
*M* *_r_* = 318.32
Monoclinic, 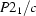

*a* = 4.488 (1) Å
*b* = 13.494 (2) Å
*c* = 26.089 (3) Å
β = 91.630 (2)°
*V* = 1579.3 (5) Å^3^
*Z* = 4
Mo *K*α radiation
μ = 0.10 mm^−1^
*T* = 298 K
0.20 × 0.20 × 0.18 mm

#### Data collection

Bruker SMART CCD diffractometer
Absorption correction: multi-scan (*SADABS*; Bruker, 2007 ▶) *T*_min_ = 0.980, *T*_max_ = 0.982
3429 measured reflections
3429 independent reflections
1584 reflections with *I* > 2σ(*I*)

#### Refinement

*R*[*F* ^2^ > 2σ(*F* ^2^)] = 0.057
*wR*(*F* ^2^) = 0.136
*S* = 0.82
3429 reflections
211 parameters
H-atom parameters constrained
Δρ_max_ = 0.17 e Å^−3^
Δρ_min_ = −0.19 e Å^−3^

### 

Data collection: *SMART* (Bruker, 2007 ▶); cell refinement: *SAINT* (Bruker, 2007 ▶); data reduction: *SAINT*; program(s) used to solve structure: *SHELXTL* (Sheldrick, 2008 ▶); program(s) used to refine structure: *SHELXTL*; molecular graphics: *SHELXTL*; software used to prepare material for publication: *SHELXTL*.

## Supplementary Material

Crystal structure: contains datablocks global, I. DOI: 10.1107/S1600536810016855/hb5423sup1.cif

Structure factors: contains datablocks I. DOI: 10.1107/S1600536810016855/hb5423Isup2.hkl

Additional supplementary materials:  crystallographic information; 3D view; checkCIF report

## Figures and Tables

**Table 1:** Hydrogen-bond geometry (Å, °)

*D*—H⋯*A*	*D*—H	H⋯*A*	*D*⋯*A*	*D*—H⋯*A*
O4—H4⋯O3	0.82	1.80	2.528 (2)	148
O1—H1⋯N1	0.82	1.93	2.650 (2)	146
N2—H2⋯O5	0.90	2.01	2.899 (2)	172
O5—H5*B*⋯O3^i^	0.84	1.92	2.745 (2)	167
O5—H5*A*⋯O1^ii^	0.84	2.06	2.849 (3)	156

Symmetry codes: (i) 
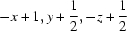
; (ii) 
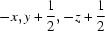
.

**Fig. 1.**
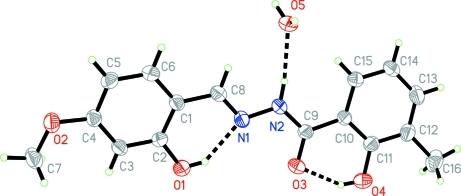
The molecular structure of (I) with 30% probability displacement ellipsoids for non-H atoms. Intramolecular hydrogen bonds are shown as dashed lines.

**Fig. 2.**
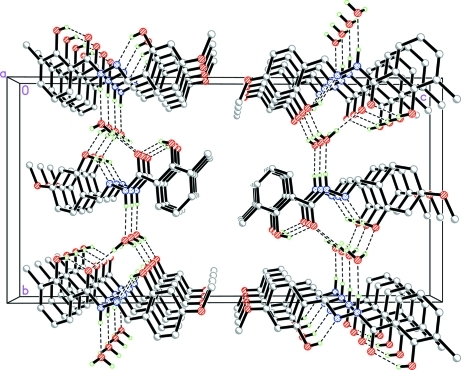
The crystal packing of (I). Hydrogen bonds are shown as dashed lines.

## supplementary crystallographic information

### Comment

As a continuation of our work on the structural characterization of hydrazones (Han & Zhao, 2010a,b), we repoprt here the crystal structure of the title compound. The title compound, Fig. 1, consists of a hydrazone molecule and a water molecule of crystallization. The dihedral angle between the two benzene rings is 12.4 (2)°. The molecule adopts an *E* configuration with respect to the C═N bond. There are intramolecular O–H···N and O–H···O hydrogen bonds in the hydrazone molecule (Table 1). All the bond lengths are within normal ranges (Allen *et al.*, 1987).

In the crystal structure, molecules are linked through intermolecular N–H···O and O–H···O hydrogen bonds (Table 1) to form layers parallel to the *ab* plane (Fig. 2).

### Experimental

A mixture of 4-methoxysalicylaldehyde (0.152 g, 1 mmol) and 2-hydroxy-3-methylbenzohydrazide (0.166 g, 1 mmol) in 50 ml me thanol was stirred at room temperature for 1 h. The mixture was filtered to remove impurities, and then left at room temperature. After a few days, colourless blocks of (I) were formed.

### Refinement

The crystal turned out to be a non-merohedral twin (twin law: -1 0 0/0 -1 0/ 0.331 0 1) with a fractional contribution of the minor component of 0.113 (3). Amino H and water H atoms were located from a difference Fourier map and refined isotropically, with N–H, O–H, and H···H distances restrained to 0.90 (1), 0.85 (1), and 1.37 (2) Å, respectively. Other H atoms were positioned geometrically and refined using the riding-model approximation, with C–H = 0.93 or 0.96 Å, O–H = 0.82 Å, and U_iso_(H) = 1.2U_eq_(C) or U_iso_(H) = 1.5U_eq_(methyl C and O).

### Crystal data

**Table:**

C_16_H_16_N_2_O_4_·H_2_O	*F*(000) = 672
*M**_r_* = 318.32	*D*_x_ = 1.339 Mg m^−^^3^
Monoclinic, *P*2_1_/*c*	Mo *K*α radiation, λ = 0.71073 Å
Hall symbol: -P 2ybc	Cell parameters from 1505 reflections
*a* = 4.488 (1) Å	θ = 2.7–24.3°
*b* = 13.494 (2) Å	µ = 0.10 mm^−^^1^
*c* = 26.089 (3) Å	*T* = 298 K
β = 91.630 (2)°	Block, colorless
*V* = 1579.3 (5) Å^3^	0.20 × 0.20 × 0.18 mm
*Z* = 4	

### Data collection

**Table:**

Bruker SMART CCD diffractometer	3429 independent reflections
Radiation source: fine-focus sealed tube	1584 reflections with *I* > 2σ(*I*)
graphite	*R*_int_ = 0.0000
ω scans	θ_max_ = 27.0°, θ_min_ = 1.6°
Absorption correction: multi-scan (*SADABS*; Bruker, 2007)	*h* = −5→5
*T*_min_ = 0.980, *T*_max_ = 0.982	*k* = −17→17
3429 measured reflections	*l* = 0→33

### Refinement

**Table:**

Refinement on *F*^2^	Primary atom site location: structure-invariant direct methods
Least-squares matrix: full	Secondary atom site location: difference Fourier map
*R*[*F*^2^ > 2σ(*F*^2^)] = 0.057	Hydrogen site location: inferred from neighbouring sites
*wR*(*F*^2^) = 0.136	H-atom parameters constrained
*S* = 0.82	*w* = 1/[σ^2^(*F*_o_^2^) + (0.0467*P*)^2^] where *P* = (*F*_o_^2^ + 2*F*_c_^2^)/3
3429 reflections	(Δ/σ)_max_ < 0.001
211 parameters	Δρ_max_ = 0.17 e Å^−^^3^
0 restraints	Δρ_min_ = −0.19 e Å^−^^3^

### Special details

**Table:**

Geometry. All esds (except the esd in the dihedral angle between two l.s. planes) are estimated using the full covariance matrix. The cell esds are taken into account individually in the estimation of esds in distances, angles and torsion angles; correlations between esds in cell parameters are only used when they are defined by crystal symmetry. An approximate (isotropic) treatment of cell esds is used for estimating esds involving l.s. planes.
Refinement. Refinement of *F*^2^ against ALL reflections. The weighted *R*-factor wR and goodness of fit *S* are based on *F*^2^, conventional *R*-factors *R* are based on *F*, with *F* set to zero for negative *F*^2^. The threshold expression of *F*^2^ > σ(*F*^2^) is used only for calculating *R*-factors(gt) etc. and is not relevant to the choice of reflections for refinement. *R*-factors based on *F*^2^ are statistically about twice as large as those based on *F*, and *R*- factors based on ALL data will be even larger.

### Fractional atomic coordinates and isotropic or equivalent isotropic displacement parameters (Å^2^)

**Table:**

	*x*	*y*	*z*	*U*_iso_*/*U*_eq_	
O1	−0.0603 (4)	0.34424 (11)	0.17742 (7)	0.0707 (5)	
H1	0.0416	0.3619	0.2024	0.106*	
O2	−0.7266 (4)	0.47988 (12)	0.05036 (6)	0.0682 (5)	
O3	0.4757 (4)	0.34626 (11)	0.30448 (6)	0.0750 (6)	
O4	0.8037 (4)	0.30326 (11)	0.38168 (7)	0.0774 (6)	
H4	0.6857	0.2940	0.3575	0.116*	
N1	0.2277 (4)	0.47228 (14)	0.23782 (7)	0.0548 (5)	
N2	0.4287 (4)	0.50268 (14)	0.27598 (7)	0.0529 (5)	
H2	0.4617	0.5681	0.2779	0.063*	
C1	−0.1155 (5)	0.52186 (16)	0.17115 (8)	0.0501 (6)	
C2	−0.1891 (5)	0.42542 (16)	0.15477 (8)	0.0507 (6)	
C3	−0.3910 (5)	0.40878 (16)	0.11519 (8)	0.0528 (6)	
H3	−0.4381	0.3443	0.1053	0.063*	
C4	−0.5233 (5)	0.48799 (16)	0.09027 (8)	0.0516 (6)	
C5	−0.4595 (6)	0.58423 (17)	0.10571 (9)	0.0579 (6)	
H5	−0.5539	0.6375	0.0895	0.069*	
C6	−0.2570 (5)	0.60000 (17)	0.14486 (9)	0.0571 (7)	
H6	−0.2116	0.6648	0.1544	0.069*	
C7	−0.7804 (7)	0.38437 (19)	0.02939 (10)	0.0779 (9)	
H7A	−0.5970	0.3571	0.0177	0.117*	
H7B	−0.9211	0.3894	0.0011	0.117*	
H7C	−0.8597	0.3420	0.0552	0.117*	
C8	0.0983 (5)	0.54235 (18)	0.21216 (9)	0.0533 (6)	
H8	0.1437	0.6078	0.2203	0.064*	
C9	0.5468 (6)	0.43567 (17)	0.30853 (9)	0.0527 (6)	
C10	0.7606 (5)	0.46971 (15)	0.34903 (8)	0.0482 (6)	
C11	0.8747 (6)	0.40063 (16)	0.38452 (9)	0.0560 (6)	
C12	1.0734 (6)	0.42876 (19)	0.42418 (9)	0.0626 (7)	
C13	1.1545 (6)	0.52613 (19)	0.42727 (10)	0.0713 (8)	
H13	1.2865	0.5462	0.4534	0.086*	
C14	1.0462 (6)	0.59542 (18)	0.39284 (10)	0.0693 (8)	
H14	1.1057	0.6612	0.3959	0.083*	
C15	0.8526 (6)	0.56823 (17)	0.35429 (9)	0.0586 (7)	
H15	0.7808	0.6157	0.3312	0.070*	
C16	1.1935 (7)	0.3513 (2)	0.46131 (10)	0.0915 (10)	
H16A	1.3286	0.3820	0.4857	0.137*	
H16B	1.2969	0.3011	0.4428	0.137*	
H16C	1.0313	0.3218	0.4790	0.137*	
O5	0.4807 (5)	0.71679 (11)	0.27610 (6)	0.0787 (6)	
H5A	0.3414	0.7383	0.2940	0.118*	
H5B	0.5224	0.7569	0.2528	0.118*	

### Atomic displacement parameters (Å^2^)

**Table:**

	*U*^11^	*U*^22^	*U*^33^	*U*^12^	*U*^13^	*U*^23^
O1	0.0818 (13)	0.0494 (10)	0.0792 (12)	−0.0028 (9)	−0.0243 (10)	0.0088 (8)
O2	0.0772 (13)	0.0656 (11)	0.0607 (10)	0.0033 (10)	−0.0179 (10)	−0.0049 (8)
O3	0.1079 (16)	0.0447 (10)	0.0717 (12)	−0.0132 (10)	−0.0130 (10)	−0.0080 (8)
O4	0.1079 (16)	0.0432 (10)	0.0804 (13)	0.0016 (10)	−0.0083 (11)	0.0055 (8)
N1	0.0563 (13)	0.0572 (12)	0.0505 (11)	−0.0060 (10)	−0.0044 (10)	−0.0047 (10)
N2	0.0592 (13)	0.0466 (10)	0.0524 (12)	−0.0060 (10)	−0.0057 (10)	−0.0047 (9)
C1	0.0540 (15)	0.0509 (14)	0.0454 (13)	−0.0051 (12)	0.0009 (12)	−0.0036 (11)
C2	0.0554 (16)	0.0456 (13)	0.0512 (13)	−0.0011 (12)	0.0017 (12)	0.0048 (11)
C3	0.0545 (15)	0.0468 (13)	0.0568 (15)	−0.0027 (12)	−0.0045 (12)	−0.0056 (11)
C4	0.0558 (15)	0.0541 (14)	0.0447 (13)	0.0000 (12)	−0.0014 (12)	−0.0044 (11)
C5	0.0660 (17)	0.0483 (13)	0.0591 (15)	0.0080 (13)	−0.0030 (13)	0.0046 (11)
C6	0.0646 (17)	0.0449 (13)	0.0616 (16)	−0.0051 (12)	0.0000 (14)	−0.0026 (11)
C7	0.086 (2)	0.0762 (19)	0.0699 (18)	−0.0058 (17)	−0.0192 (15)	−0.0202 (15)
C8	0.0521 (15)	0.0521 (14)	0.0556 (14)	−0.0075 (12)	0.0005 (12)	−0.0052 (12)
C9	0.0694 (17)	0.0417 (13)	0.0470 (13)	−0.0014 (12)	0.0046 (12)	−0.0046 (11)
C10	0.0573 (15)	0.0429 (12)	0.0445 (13)	0.0017 (11)	0.0012 (11)	−0.0044 (10)
C11	0.0699 (18)	0.0465 (14)	0.0518 (14)	0.0044 (13)	0.0070 (13)	−0.0021 (11)
C12	0.0727 (19)	0.0635 (16)	0.0515 (15)	0.0114 (15)	−0.0028 (13)	0.0005 (13)
C13	0.078 (2)	0.0724 (18)	0.0620 (17)	0.0062 (16)	−0.0158 (15)	−0.0129 (14)
C14	0.086 (2)	0.0499 (14)	0.0707 (18)	−0.0079 (14)	−0.0158 (16)	−0.0095 (13)
C15	0.0698 (18)	0.0456 (13)	0.0601 (15)	0.0024 (13)	−0.0052 (13)	−0.0020 (11)
C16	0.112 (3)	0.092 (2)	0.0700 (19)	0.034 (2)	−0.0149 (17)	0.0079 (16)
O5	0.1172 (17)	0.0435 (9)	0.0748 (12)	0.0068 (10)	−0.0079 (11)	0.0063 (8)

### Geometric parameters (Å, °)

**Table:**

O1—C2	1.365 (3)	C7—H7A	0.9600
O1—H1	0.8208	C7—H7B	0.9600
O2—C4	1.369 (3)	C7—H7C	0.9600
O2—C7	1.418 (3)	C8—H8	0.9300
O3—C9	1.252 (3)	C9—C10	1.480 (3)
O4—C11	1.354 (3)	C10—C15	1.398 (3)
O4—H4	0.8210	C10—C11	1.400 (3)
N1—C8	1.287 (3)	C11—C12	1.399 (3)
N1—N2	1.387 (2)	C12—C13	1.365 (3)
N2—C9	1.339 (3)	C12—C16	1.514 (3)
N2—H2	0.8961	C13—C14	1.375 (3)
C1—C6	1.400 (3)	C13—H13	0.9300
C1—C2	1.406 (3)	C14—C15	1.361 (3)
C1—C8	1.443 (3)	C14—H14	0.9300
C2—C3	1.373 (3)	C15—H15	0.9300
C3—C4	1.377 (3)	C16—H16A	0.9600
C3—H3	0.9300	C16—H16B	0.9600
C4—C5	1.387 (3)	C16—H16C	0.9600
C5—C6	1.364 (3)	O5—H5A	0.8417
C5—H5	0.9300	O5—H5B	0.8395
C6—H6	0.9300		
			
C2—O1—H1	109.3	N1—C8—C1	121.7 (2)
C4—O2—C7	117.94 (18)	N1—C8—H8	119.2
C11—O4—H4	109.6	C1—C8—H8	119.2
C8—N1—N2	115.52 (18)	O3—C9—N2	120.1 (2)
C9—N2—N1	119.55 (19)	O3—C9—C10	121.2 (2)
C9—N2—H2	124.6	N2—C9—C10	118.68 (19)
N1—N2—H2	115.8	C15—C10—C11	117.8 (2)
C6—C1—C2	116.7 (2)	C15—C10—C9	123.3 (2)
C6—C1—C8	120.1 (2)	C11—C10—C9	118.9 (2)
C2—C1—C8	123.2 (2)	O4—C11—C12	116.6 (2)
O1—C2—C3	117.17 (19)	O4—C11—C10	121.9 (2)
O1—C2—C1	121.21 (19)	C12—C11—C10	121.4 (2)
C3—C2—C1	121.6 (2)	C13—C12—C11	117.9 (2)
C2—C3—C4	119.7 (2)	C13—C12—C16	122.5 (2)
C2—C3—H3	120.2	C11—C12—C16	119.6 (2)
C4—C3—H3	120.2	C12—C13—C14	121.8 (2)
O2—C4—C3	124.5 (2)	C12—C13—H13	119.1
O2—C4—C5	115.0 (2)	C14—C13—H13	119.1
C3—C4—C5	120.5 (2)	C15—C14—C13	120.4 (2)
C6—C5—C4	119.4 (2)	C15—C14—H14	119.8
C6—C5—H5	120.3	C13—C14—H14	119.8
C4—C5—H5	120.3	C14—C15—C10	120.7 (2)
C5—C6—C1	122.2 (2)	C14—C15—H15	119.7
C5—C6—H6	118.9	C10—C15—H15	119.7
C1—C6—H6	118.9	C12—C16—H16A	109.5
O2—C7—H7A	109.5	C12—C16—H16B	109.5
O2—C7—H7B	109.5	H16A—C16—H16B	109.5
H7A—C7—H7B	109.5	C12—C16—H16C	109.5
O2—C7—H7C	109.5	H16A—C16—H16C	109.5
H7A—C7—H7C	109.5	H16B—C16—H16C	109.5
H7B—C7—H7C	109.5	H5A—O5—H5B	111.4
			
C8—N1—N2—C9	172.1 (2)	N1—N2—C9—C10	−179.92 (19)
C6—C1—C2—O1	179.1 (2)	O3—C9—C10—C15	178.3 (2)
C8—C1—C2—O1	0.3 (4)	N2—C9—C10—C15	−1.8 (4)
C6—C1—C2—C3	−0.4 (4)	O3—C9—C10—C11	−2.6 (4)
C8—C1—C2—C3	−179.2 (2)	N2—C9—C10—C11	177.2 (2)
O1—C2—C3—C4	−178.7 (2)	C15—C10—C11—O4	−178.4 (2)
C1—C2—C3—C4	0.8 (4)	C9—C10—C11—O4	2.5 (4)
C7—O2—C4—C3	−7.4 (4)	C15—C10—C11—C12	0.3 (4)
C7—O2—C4—C5	174.0 (2)	C9—C10—C11—C12	−178.9 (2)
C2—C3—C4—O2	179.9 (2)	O4—C11—C12—C13	178.5 (3)
C2—C3—C4—C5	−1.6 (4)	C10—C11—C12—C13	−0.2 (4)
O2—C4—C5—C6	−179.3 (2)	O4—C11—C12—C16	−0.6 (4)
C3—C4—C5—C6	2.0 (4)	C10—C11—C12—C16	−179.3 (3)
C4—C5—C6—C1	−1.7 (4)	C11—C12—C13—C14	0.1 (4)
C2—C1—C6—C5	0.8 (4)	C16—C12—C13—C14	179.2 (3)
C8—C1—C6—C5	179.7 (2)	C12—C13—C14—C15	0.0 (5)
N2—N1—C8—C1	179.8 (2)	C13—C14—C15—C10	0.0 (4)
C6—C1—C8—N1	178.5 (2)	C11—C10—C15—C14	−0.2 (4)
C2—C1—C8—N1	−2.7 (4)	C9—C10—C15—C14	178.9 (2)
N1—N2—C9—O3	−0.1 (4)		

### Hydrogen-bond geometry (Å, °)

**Table:**

*D*—H···*A*	*D*—H	H···*A*	*D*···*A*	*D*—H···*A*
O4—H4···O3	0.82	1.80	2.528 (2)	148
O1—H1···N1	0.82	1.93	2.650 (2)	146
N2—H2···O5	0.90	2.01	2.899 (2)	172
O5—H5B···O3^i^	0.84	1.92	2.745 (2)	167
O5—H5A···O1^ii^	0.84	2.06	2.849 (3)	156

Symmetry codes: (i) −*x*+1, *y*+1/2, −*z*+1/2; (ii) −*x*, *y*+1/2, −*z*+1/2.
